# A descriptive study of percutaneous injuries in National Healthcare Group POLYCLINICS dental clinics in Singapore from 2014 to 2020

**DOI:** 10.1038/s41405-023-00171-7

**Published:** 2023-10-16

**Authors:** Vivian Yung Yee Wong, Priscilla Jang Shing Chao, Sabrina Poay Sian Lee, Eng Sing Lee, Lily Ren Lee Lang, Holy JR Koh, Kenneth Meng Tze Low

**Affiliations:** National Healthcare Group Polyclinics Singapore, Singapore, Singapore

**Keywords:** Occupational health, Infection control in dentistry

## Abstract

**Introduction:**

All dental staff face risk of percutaneous injuries (PCI)s. Blood-borne diseases may be transmitted to staff via contaminated sharp instruments. Hence there are significant impacts on staff when PCIs occur. Though a PCI is an occupational hazard, it is preventable.

**Aim:**

This study aims to identify factors associated with PCIs among dental staff by evaluating the circumstances and staff designations involved.

**Methods:**

PCIs were reported through an electronic incident reporting system from 2014 to 2020. Reports involved their nature and extent. Statistical analysis was carried out to find associations between factors such as injury site, type of instrument and staff designation.

**Results:**

A total of 63 PCIs were included in this study. The type of instrument was found to be significantly associated with staff designation (*p* = 0.04, *p* < 0.05) with significantly more dental burs causing injury in dentists and more injuries caused by ‘other instruments’ in health attendants (*p* = 0.0083). Majority of PCIs occurred in dentists, then dental assistants and health attendants. Staff designation was significantly associated with the instance where PCIs occurred (*p* < 0.001). Dentists and dental assistants were more likely to sustain injuries during a dental procedure than before procedure and after procedure (*p* = 0.0167). The mean incidence of PCIs among our dentists was 15.6/100.

**Conclusions:**

All dental staff are at risk of PCIs however dentists sustain the highest number of PCIs. Needles, dental burs and metal matrices are the top three instruments. Targeted interventions might help prevent/reduce PCIs.

## Introduction

Percutaneous injuries (PCI)s are occupational hazards among healthcare workers (HCW)s. Blood-borne infectious agents such as the human immunodeficiency virus (HIV), Hepatitis B virus (HBV) and Hepatitis C virus (HCV) may be transmitted through contaminated sharp injuries [1].

Various dental professional groups face the risk of PCI, as needles and sharp instruments are common to almost all dental procedures. The risks of contracted infections after sustaining a pathogen-positive PCI are 6.0–30.0% for HBV, 0–10.0% for HCV and 0.3% for HIV [1–3]. The associated economic and psychological impact of such incidents are significant, even when a positive transmission does not occur [4–7]. For example, breaking the news to patients, time taken for staff to attend medical consultations and time taken for staff to go for repeated blood tests.

Most published data on PCI incidence rates are derived from non-dental settings. A Singapore University Hospital demonstrated incidence rates of 4.1% in 2014 [8]. While the annual incidence of PCI was found to be 8.19% among dental workers in a Taiwan University Hospital [9]. As the dental clinical set up differs from other medical specialties, it would be important to look specifically at data from dental settings when developing prevention strategies.

The National Healthcare Group Polyclinics (NHGP) Dental Services serves patients in Singapore in a primary care setting. Four dental clinics serve a significant proportion of the Singapore population in the central and northern parts of Singapore.

This study aims to identify the factors related to PCI at our dental polyclinics from 2014 to 2020. These factors are, staff category involved in PCI, day of the week and time of the day of the occurrences, devices that are involved, severity of injury, site of injury, staff length of experience and if it was a clean or contaminated injury. The secondary aim was to explore the associations of PCI among dental HCWs in the primary care setting. In doing so, we can find ways to prevent and target preventive efforts and improve workplace safety for dental staff. Some research questions we hope to answer are as follows: Is there any association between the ‘staff designation’ and the sharp injury caused by the ‘type of device’? Do PCIs occur more often when it is closer to lunchtime or end of work or a certain day of week? Does the ‘type of device’ have any association with ‘severity of injury’ or ‘site of injury’? Is the ‘staff designation’ related to the instance where the sharp incident occurs? Are clinicians with a shorter length of service more prone to PCIs? Do clean injuries occur more frequently in a particular staff designation?

## Materials and methods

This was a retrospective study with data collected from an electronic incident reporting system from 2014 to 2020. Ethical approval was sought from the National Healthcare Group Domain Specific Review Board (DSRB) Singapore (study reference 2021/00428).

### Study population

All dental staff who had sustained a PCI and had submitted an incident report into the electronic incident reporting system (within 24 h from injury) from January 2014 to December 2020 were included in the study. This included all dental staff from four dental clinics which consisted of dentists, oral health therapists, dental assistants and health attendants. There was no sample size calculation as this was an observational study within a fixed time period.

### Study schedule

This study started in July 2021. The data collection period was from July 2021 to September 2021. Data was collated from October 2021 to Dec 2021 and were then analysed from Jan 2022 to June 2022.

### Inclusion criteria

The inclusion criteria involve all staff who had a PCI involving a sharp instrument, with an incident report submitted on the electronic incident reporting system within 24 h of injury. The protocol of managing a PCI must have been followed based on the procedure manual (Supplementary Appendix 1) where blood was drawn from staff and attended consultation with a doctor.

Supplementary Appendix 1 shows a matrix of how a PCI is managed. Following a PCI, the staff stops all work, first aid is provided to the injury by washing under running water and gently expressing the site to encourage bleeding. The staff is seen by a doctor within the polyclinic who does a risk assessment of the injury and source patient, after which a referral is made to National Centre for Infectious Diseases (NCID) according to risk status (refer to Supplementary Appendix 1) and the supervisor and nurse manager (Author Lily R.L Lang) will directly check in with the staff, track the outcome of the visit and inform of the date of the next appointment.

Blood is taken with consent from both the source patient and the injured staff and tested for Hepatitis B, Hepatitis C and HIV. The follow up visits for the injured staff are as follows: Immediately or next day on occurrence of injury, then one month, three months, six months and a year (if involving HIV positive source) after the injury is sustained.

### Exclusion criteria

We excluded body fluid splashes that did not result in PCI and this included eye splashes. Other exclusion criteria included incomplete incident report, delay in incident reporting (>24 h after the incident). And excluded those where the protocol was not followed and no blood was taken from staff.

Data was retrieved from the electronic system and anonymised by a staff independent of the research team. A serial number was assigned to the subjects involved. The principal investigator, (VYYW) and the co-investigator (PJSC) were involved with the data collection and data entry into the data collection form (Supplementary Appendix 2). As all data was retrospective and anonymised by a third party before being passed on to the research team, a waiver of consent was sought from the ethics committee.

Another co-investigator (HJK) checked the accuracy of data entered in 10% of all the subjects entered. Where there was a discrepancy in data entry, the co-investigator checked another 10% of the data entry. Any discrepancies were discussed with the research team.

Data on the nature of the injury, staff job title, length of service, site of injury, extent (deep/moderate/shallow injury), day of the week, frequency of injury to a particular staff (e.g. first occurrence etc.) and devices involved in the PCI were collected. Data on the number of staff employed, the total patient attendances per year per clinic and the total procedures done per year per clinic were collected to provide context for the study setting.

### Definitions

In this study, a contaminated injury was defined as an injury caused by an item that has come in contact with blood or body fluid. A clean injury was defined as an injury caused by an item that did not contact blood or body fluids or has gone through a physical or chemical means where blood borne pathogens were no longer capable of transmitting infectious particles and was rendered safe for use. A clean item causing injury through a contaminated glove was also considered a contaminated injury.

The severity of injury was defined. A shallow injury was one with minor/superficial with no or minimal bleeding. A moderate injury was one that perforated the skin with bleeding. A deep injury was defined as a through and through injury or extensive bleeding.

### Statistical analysis

All statistical analysis was conducted using IBM SPSS Statistics for Windows, Version 22.0 (Armonk, NY: IBM Corp). Descriptive statistics was used to describe the factors related to the PCI. Fisher exact test, Bonferroni post-hoc test and Poisson regression were used to investigate associations between factors.

## Results

### Descriptive statistics

There were a total of 66 PCIs during the study period. 3 were excluded due to missing data and none due to delayed reporting. Hence the total number of PCIs included in study were 63 (see Table 1 for descriptive statistics).

**Table 1:** Descriptive statistics of PCI from January 2014 to December 2020 (*N* = 63).

Factor	Number of injuries *N*. (%)
Year	
2014	10 (15.9%)
2015	11 (17.5%)
2016	6 (9.5%)
2017	11 (17.5%)
2018	10 (15.9%)
2019	8 (12.7%)
2020	7 (11.1%)
Staff designation	
Dentist	47 (74.6%)
Dental assistant	12 (19.0%)
Health attendant	3 (4.8%)
Oral health therapist	1 (1.6%)
Clinic (anonymised)	
Clinic 1	26 (41.3%)
Clinic 2	15 (23.8%)
Clinic 3	13 (20.6%)
Clinic 4	9 (14.3%)
Gender	
Male	11 (17.5%)
Female	52 (82.5%)
Length of experience of staff	
<1 month	11 (17.5%)
1–3 months	11 (17.5%)
3–6 months	15 (23.8%)
>6 months	26 (41.3%)
Instance of incident	
Before start of procedure	1 (1.6%)
During procedure	51 (80.9%)
After procedure (cleanup)	11 (17.5%)
Clean or contaminated?	
Clean	4 (6.3%)
Contaminated	59 (88.9%)
Type of device	
Dental bur	15 (23.8%)
Extraction instruments	5 (7.9%)
Metal matrix band	10 (15.9%)
Needle (LA needle and suture needle)	20 (31.7%), 14 (22.2%) were LA needles and 6 (9.5%) were suture needles.
Scaler tip	6 (9.5%)
Other instruments (dental probe, spoon excavator, endodontic file, rubber dam clamp, patient’s bite/teeth)	7 (11.1%)
Site of injury	
Left hand	29 (46.0%)
Right hand	33 (52.4%)
Left thigh	1 (1.6%)
Severity of injury	
Shallow	47 (74.6%)
Moderate	16 (25.4%)
Deep	0 (0%)
Day of week	
Monday	9 (14.3%)
Tuesday	12 (19.0%)
Wednesday	14 (22.2%)
Thursday	13 (20.6%)
Friday	10 (15.9%)
Saturday	5 (7.9%)

Out of the total 63 PCIs that occurred during the study period, the majority occurred among the dentists (74.6%) followed by dental assistants (19%), followed by health attendants (4.8%) and oral health therapists (1.6%). In the 7-year study period, the average number of PCIs per 100,000 patient attendances per year was 9.22 (range, 6.48–12.86) [Table 2]. The average number of PCIs per 100,000 procedures per year was 3.68 (range, 2.39–4.62) [Table 3].

**Table 2:** Number of patient attendances per year per clinic.

		Attendance					
Number of PCI	Year	Clinic 4	Clinic 3	Clinic 2	Clinic 1	Total	Number of PCI per 100000 patient attendances
10	2014	14,066	26,022	28,242	28,412	96,742	10.34
11	2015	13,426	30,448	29,916	29,006	102,796	10.70
6	2016	13,874	31,181	29,902	32,208	107,165	5.60
11	2017	14,113	31,438	30,899	34,773	111,223	9.89
10	2018	20,177	28,592	27,549	39,092	115,410	8.66
8	2019	25,807	29,216	27,236	41,125	123,384	6.48
7	2020	12,673	11,943	11,404	18,396	54,416	12.86
	Total sum	114,136	188,840	185,148	223,012	711,136	Average 9.22

**Table 3:** Number of procedures done per year per clinic.

		No. of Procedures					
Number of PCI	Year	Clinic 4	Clinic 3	Clinic 2	Clinic 1	Total	Number of PCI per 100000 procedures
10	2014	34,152	58,325	63,093	68,197	223,767	4.47
11	2015	32,046	66,613	69,078	70,291	238,028	4.62
6	2016	32,798	67,313	71,650	79,303	251,064	2.39
11	2017	33,696	70,891	76,470	86,799	267,856	4.11
10	2018	52,401	70,599	70,152	98,239	291,391	3.43
8	2019	68,137	76,890	75,577	112,213	332,817	2.40
7	2020	38,547	38,506	35,334	49,567	161,954	4.32
	Total Sum	291,777	449,137	461,354	564,609	1,766,877	Average = 3.68

80.9% of PCIs occurred during the dental procedure followed by 17.5% occurring after procedure (during the cleanup) and 1.6% occurred before procedure (during preparation).

The top three dental devices or instruments that were related to the PCIs were the dental bur (23.8%) followed by the LA needle (22.2%) and metal matrix band (15.9%).

Majority of PCIs occurred on the right hand (52.4%) compared to the left hand (46.0%).

### Analysis of PCIs

**Fig. 1:**
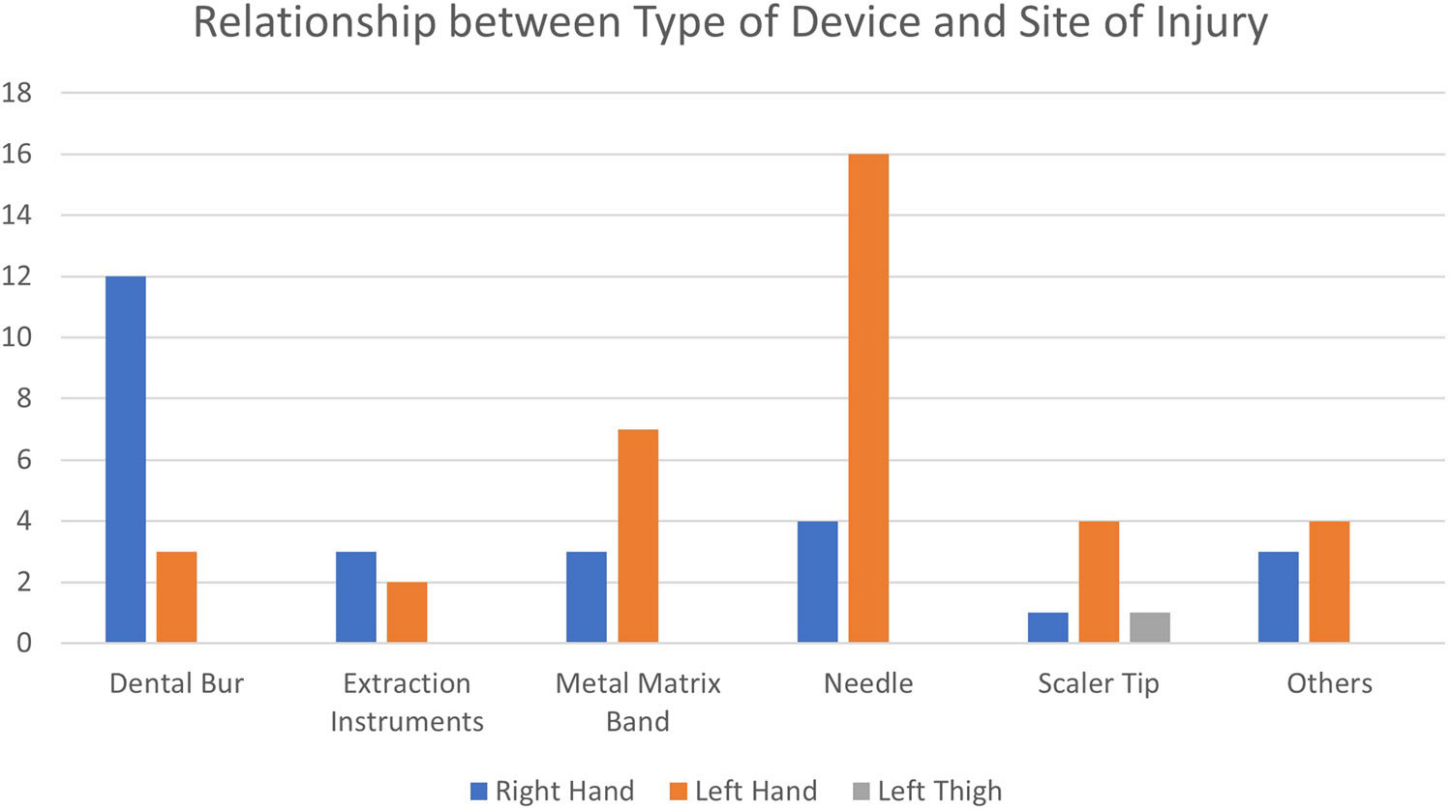
Bar Chart showing relationship between type of device/instrument and site of injury.

Using the Fisher’s exact test, the type of device was found to be significantly associated with staff designation (*p* = 0.04, *p* < 0.05) and significantly associated with the site of injury (*p* = 0.002). Bonferroni post-hoc tests revealed that there were significantly more dental burs causing injuries in dentists and more injuries caused by ‘other instruments’ in health attendants (*p* = 0.0083); and there were statistically significantly more needle injuries in the left hand than right hand (*p* < 0.05) [Fig. 1]. However, the type of device was not significantly associated with the severity of injury (*p* = 0.93).

Using the Fisher’s exact test, staff designation was significantly associated with the instance where the sharp incident occurred (*p* < 0.001), also PCIs were not significantly associated with a certain time of the day (*p* = 0.47, *p* > 0.05) or day of the week (*p* = 0.44, *p* > 0.05). The frequency of clean injuries was not significantly associated with a particular staff grade (*p* = 0.05). Bonferroni post-hoc tests revealed that there were statistically significantly more dentists and dental assistants sustaining injuries during a dental procedure than before procedure and after procedure (clean up) (*p* = 0.0167). A Poisson regression was run to predict the tendency of PCI based on clinicians’ length of service. Longer length of service was significantly associated with higher tendency for sharp injuries (*p* = 0.03).

From following up on the incidences that occurred from 2014 to 2020, the author Lily Lang R.L confirmed that there was an absence of blood borne infections among staff. There was no staff who suffered multiple PCIs.

## Discussion

An infection control policy was put in place within our dental clinics in 2012 which included safe sharp handling practices, monthly audits and instructions to report, through an electronic incident reporting system within 24 h of a PCI. Dental HCWs must comply with these however no matter how careful they are, PCIs still occur.

The average annual number of PCIs in dental HCWs in our study ranged from 6 to 11 a year, similar to that found by Iwamatsu-Kobayashi et al. [10] in Japan, although the dental setting was university hospitals. The approximate annual incidence in our study of 8.4 per 100 staff [Table 4] seems similar that found in a university hospital in Taiwan [9]. Some studies reported that underreporting is a common problem [11, 12]. However, as our dental clinics have workers’ compensation insurance covering PCIs, and insurance coverage will only be given with reporting of injuries, underreporting may be less prevalent in our study.

**Table 4:** Number of staff working in NHGP from 2014-2020.

Year	Total dentists	Total oral health therapists	Total dental assistants	Total health attendants	Dentist PCI	Incidence rate for dentists	Mean incidence for dentists
2014	37	6	42	13	7	18.92	15.56
2015	42	5	43	12	10	23.81	
2016	43	5	45	13	5	11.63	
2017	43	5	48	12	9	20.93	
2018	47	8	53	13	6	12.77	
2019	51	6	58	13	4	7.84	
2020	46	4	54	14	6	13.04	

Mean staff strength/year = 111.5.

Mean incidence for all staff = [(66/7)/111.5] × 100 = 8.4 per 100.

In the local context, the incidence of PCIs among our dentists in the primary care setting ranged between 7.8/100 to 23.8/100 across 7 years, with a mean incidence of 15.6/100 [Table 4]. This is lower compared to 21.3/100 reported for doctors in a university hospital in 2014 [8].

### Type of device

Needles and dental burs were the commonest devices causing PCIs among dentists. This was consistent with other studies done in non-hospital settings [13, 14]. The third commonest device involved with PCI was the metal matrix band. This is not surprising, as placing fillings form the bulk of general dentistry. Majority of injuries occurred on the right hand compared to the left because most of our staff were right-handed. Most of our dental chairs have handpieces placed in the dentist element and may graze or prick their right hand while reaching for instruments. This was similarly observed in another study by Iwamatsu-Kobayashi et al. [10] where PCIs occurred more commonly during a dental procedure rather than before or after treatment. This was the time where most sharps instruments were handled.

A high number of needle injuries were associated with the left hand, which was significant but this was not caused by recapping of the needle using both hands. When looking into how the injuries occurred, some causes include during assembly/removal of the LA cartridge when changing cartridge, retraction of the lips using the left finger during suturing or administering LA, failure to screw the needle onto the syringe properly hence the needle disassembled and caused a prick on the non-dominant left hand.

Majority of the PCIs in our study were shallow injuries, which involved minimal or no bleeding, with no PCIs causing deep injuries. We postulated an association between the type of device used and how severe the injury was. However, the type of device was not found to be significantly associated with the severity of injury. This was a good finding as it meant minimal harm was involved with a PCI in the dental setting.

### Time of the day/day of the week

No significant findings were found in relation to the time of the day or day of the week. We were interested to find out if more PCIs occurred after midday/mid-week or end of the day/end of the week where one would be more tired and lacked focus. However, this was not found to be the case. However, in other studies, on the working days in the middle (Wednesday) and end (Friday) of the week, and at the hours close to lunch break (11:00 to 14:00) and getting off duty (after 16:00), there tended to be more PCIs [9].

### Clean or contaminated injuries

Frequency of clean injuries was not significantly associated with a particular staff grade (*p* = 0.05). We had initially hypothesised that perhaps staff (Health attendants) involved with sterilisation and decontamination would have more tendency to get a PCI from a clean instrument rather than a contaminated instrument. However, this was not found to be the case. This was a good finding as it meant that everyone has the same tendency for clean/contaminated PCIs and everyone should be equally vigilant in preventing PCIs.

### Staff length of service

Our study found that longer length of service was significantly associated with higher tendency for sharp injuries, *p* = 0.03. This seemed to contradict other studies where staff or students with less clinical experience had greater PCIs [9, 15, 16]. As our dental setting is different from a university hospital, the staff population in our study differs and hence the results differ. Perhaps staff who have worked longer within our clinics were less focused and attentive towards safe sharp practices. Also to note, the classification of the length of service is different from that of the years of clinical experience in the literature, hence we may not be able to conclude that PCIs occur more frequently in those with longer clinical experience. Nevertheless, it is still important to have reminders and continual education for all staff in preventive efforts.

### Preventive efforts

**Fig. 2:**
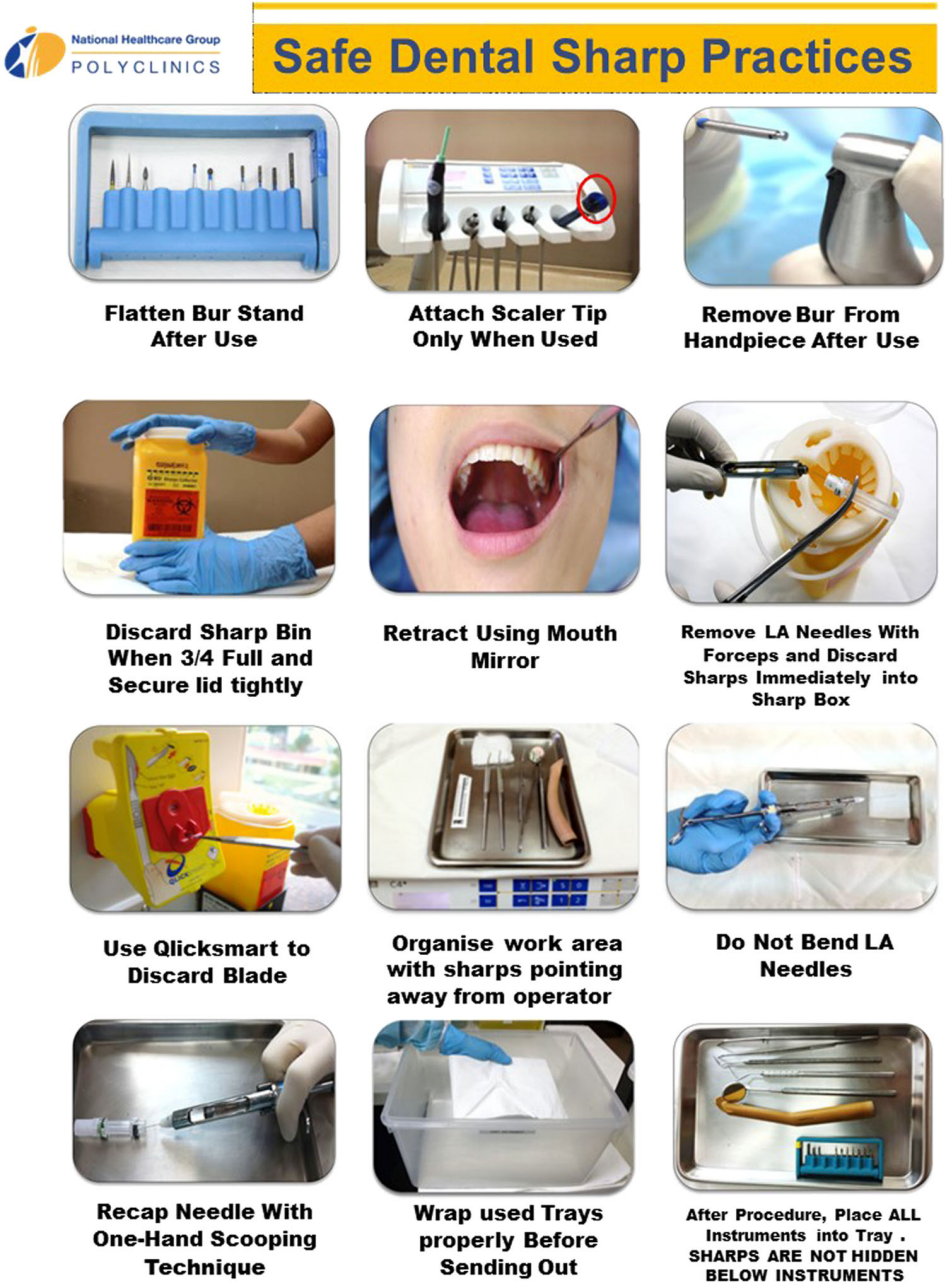
Sharps poster placed in each room. Poster created by author Lily Ren Lee Lang, used with permission from the dental department director and author Kenneth Meng Tze Low.

Following observation of the above-mentioned trends, we have targeted efforts to prevent PCI. Figure 2 shows some of the safe sharp practices that are implemented in our clinics. These practices are incorporated into dental staff orientation modules and on-the-job training when staff first joins the clinic. This is in the form of videos and demonstrations. Posters containing pictures on safe sharp practices are placed in each room. Yearly infection control competencies and monthly infection control audits incorporating safe sharp practices are also implemented.

### Limitations of study

Small sample sizes were used in the comparisons in this study hence there might be bias in the results, this was because it would make it difficult to determine if the outcomes of the statistical tests were true findings and would increase the margin of error. It may then be difficult to extrapolate the results. However, as this study was based on PCIs occurring among staff, it would not be possible to increase the sample size in the time period. This observational study can only be improved if we prospectively collect data from subsequent years. A low PCI sample size was a good reflection that our preventive efforts were working.

Due to the nature of the workflow of reporting sharps injury, all reports should be submitted on the electronic portal/recording system within 24 h of the incident occurring. To note, the author Lily Lang R. L’s (nurse manager) ensures that all sharps injuries are reported in the system within the 24-h time frame and hence none of the reports were excluded due to delayed reporting.

In addition, this was mainly a descriptive study. We are unable to determine the causes of the PCIs.

## Conclusion

Within the limitations of this study, all dental HCWs are at risk of PCIs. Dentists are particularly prone to PCIs due to the dental bur and needles. Targeted interventions might help to reduce such occurrences. All dental HCWs should practice safe handling/ cleaning of instruments to prevent PCIs.

### Supplementary information

Appendices 1-2

## Data Availability

Data is available on request from the authors. The data that support the findings of this study are available from the corresponding author (VYYW), upon reasonable request.

## References

[1] Rapiti E, Prüss-Üstün A, Hutin Y. Sharps injuries : assessing the burden of disease from sharps injuries to health-care workers at national and local levels. Geneva, World Health Organization, 2005. (WHO Environmental Burden of Disease Series, No. 11).

[2] Mengistu DA, Dirirsa G, Mati E, Ayele DM, Bayu K, Deriba W (2022). Global occupational exposure to blood and body fluids among healthcare workers: systematic review and meta-analysis. Can J Infect Dis Med Microbiol.

[3] Samaranayake L, Scully C (2013). Needlestick and occupational exposure to infections: a compendium of current guidelines. Br Dent J.

[4] Mannocci A, De Carli G, Di Bari V, Saulle R, Unim B, Nicolotti N (2016). How much do needlestick injuries cost? A systematic review of the economic evaluations of needlestick and sharps injuries among healthcare personnel. Infect Control Hosp Epidemiol.

[5] Cooke CE, Stephens JM (2017). Clinical, economic, and humanistic burden of needlestick injuries in healthcare workers. Med Devices.

[6] Sohn JW, Kim BG, Kim SH, Han C (2006). Mental health of healthcare workers who experience needlestick and sharps injuries. J Occup Health.

[7] Kunishima H, Yoshida E, Caputo J, Mikamo H (2019). Estimating the national cost burden of in-hospital needlestick injuries among healthcare workers in Japan. PLoS One.

[8] Seng M, Sng GK, Zhao X, Venkatachalam I, Salmon S, Fisher D (2016). Needlestick injuries at a tertiary teaching hospital in Singapore. Epidemiol Infect.

[9] Lee JJ, Kok SH, Cheng SJ, Lin LD, Lin CP (2014). Needlestick and sharps injuries among dental healthcare workers at a university hospital. J Formos Med Assoc.

[10] Iwamatsu-Kobayashi Y, Watanabe J, Kusama T, Endo H, Ikeda S, Tokuda K, et al. A 19-year study of dental needlestick and sharps injuries in Japan. Int Dent J. 2023;73:114–20. 10.1016/j.identj.2022.04.009 PMC9875281 35810013

[11] Pervaiz M, Gilbert R, Ali N (2018). The prevalence and underreporting of needlestick injuries among dental healthcare workers in Pakistan: a systematic review. Int J Dent.

[12] Westall JO, Dickinson C (2017). Compliance with occupational exposure risk management procedures in a dental school setting. Br Dent J.

[13] Pereira MC, Mello FW, Ribeiro DM, Porporatti AL, da Costa SJ, Flores-Mir C (2018). Prevalence of reported percutaneous injuries on dentists: a meta-analysis. J Dent.

[14] Leggat PA, Smith DR (2006). Prevalence of percutaneous exposure incidents amongst dentists in Queensland. Aust Dent J.

[15] Matsumoto H, Sunakawa M, Suda H, Izumi Y (2019). Analysis of factors related to needle-stick and sharps injuries at a dental specialty university hospital and possible prevention methods. J Oral Sci.

[16] Wicker S, Rabenau HF (2010). Occupational exposures to bloodborne viruses among German dental professionals and students in a clinical setting. Int Arch Occup Environ Health.

