# Supplementary figures and images for: A descriptive study of percutaneous injuries in National Healthcare Group POLYCLINICS dental clinics in Singapore from 2014 to 2020
Source: BDJ Open. 2023 Oct 16;9:45. doi: 10.1038/s41405-023-00171-7 (PMC10579301; doi:10.1038/s41405-023-00171-7)

## Appendix 1

### 5.0 PROCEDURE

#### Workflow for managing sharps injury

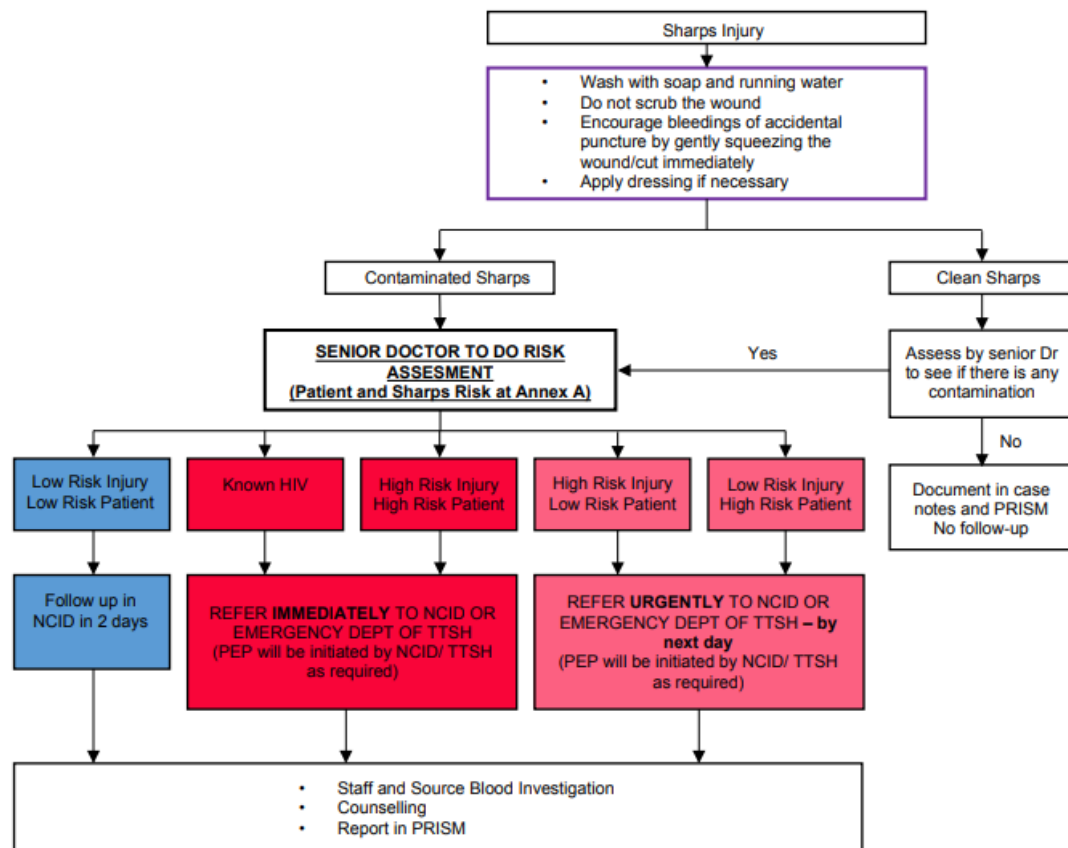

## Appendix 2

[illegible]

Supplement: Supplementary file 1 — Appendices 1-2 [file 41405_2023_171_MOESM1_ESM.pdf]
